# Effect of Hyaluronic Acid and Pluronic-F68 on the Surface Properties of Foam as a Delivery System for Polidocanol in Sclerotherapy

**DOI:** 10.3390/pharmaceutics12111039

**Published:** 2020-10-30

**Authors:** Teresa del Castillo-Santaella, Yan Yang, Inmaculada Martínez-González, María José Gálvez-Ruiz, Miguel Ángel Cabrerizo-Vílchez, Juan Antonio Holgado-Terriza, Fernando Selles-Galiana, Julia Maldonado-Valderrama

**Affiliations:** 1 Department of Applied Physics, University of Granada, Campus de Fuentenueva, sn, 18071 Granada, Spain; tdelcastillo@ugr.es (T.d.C.-S.); yan@ugr.es (Y.Y.); inmamargon97@correo.ugr.es (I.M.-G.); mjgalvez@ugr.es (M.J.G.-R.); mcabre@ugr.es (M.Á.C.-V.); 2 Excellence Research Unit “Modeling Nature” (MNat), University of Granada, 18071 Granada, Spain; 3 Department of Software Engineering, University of Granada, C/Periodista Daniel Saucedo Aranda, sn, 18071 Granada, Spain; jholgado@ugr.es; 4 Vascular Clinic Fernando Selles, C/Camino de la Zubia 27, 18006 Granada, Spain; drfernandoselles@gmail.com

**Keywords:** polidocanol, foam, sclerotherapy, hyaluronic acid, poloxamer, surface tension, surface elasticity

## Abstract

The use of foams to deliver bioactive agents and drugs is increasing in pharmaceutics. One example is the use of foam as a delivery system for polidocanol (POL) in sclerotherapy, with the addition of bioactive compounds to improve the delivery system being a current subject of study. This work shows the influence of two bioactive additives on the structure and stability of POL foam: hyaluronic acid (HA) and Pluronic-F68 (F68). HA is a natural non-surface-active biopolymer present in the extracellular matrix while F68 is a surface-active poloxamer that is biocompatible with plasma-derived fluids. Both additives increase the bulk viscosity of the sample, improving foam stability. However, HA doubled and F68 quadruplicated the foam half lifetime of POL. HA reduced the size and polydispersity of the bubble size distribution and increased the surface elasticity with respect to POL. Both facts have a positive impact in terms of foam stability. F68 also altered bubble structure and increased surface elasticity, again contributing to the enhancement of foam stability. The surface characterization of these systems is important, as in foam sclerotherapy it is crucial to assure the presence of POL at the surface of the bubbles in order to deliver the sclerosant agent in the target vein.

## 1. Introduction

Application of biotechnological tools to biomedicine is an emerging area of research. Understanding the physicochemical properties of new delivery systems allows for improvement of the transport, targeting, and controlled release of compounds at the site of action. One of the major applications of nanotechnology in medicine and pharmaceutics is the use of nanocarriers as drug delivery systems [1]. Nanocarriers can provide a vehicle for poorly soluble drugs, overcoming biological barriers to reach diseased areas. In addition, the surface properties of these carriers can be tuned to improve their performance, especially in targeted delivery. Different types of nanocarriers include micelles, liposomes, nanoparticles, emulsions, and foams [2].

The use of foam technology to deliver a range of bioactive agents and drugs is increasing in pharmaceutics, especially for topical applications [3]. The use of foam drug delivery systems applies to anti-inflammatory, anesthetics, protectives, and antiseptics, enhancing the effectiveness of the therapy by instant absorption [4]. In the last years, foams are being applied in treatment of varicose veins as delivery systems for sclerotic drugs [5]. Varicose veins are formed by malfunctioning of vein valves causing blood reflux and swollen veins with blue or purple appearance in the legs. Sclerotherapy consists in the injection of sclerosing agents directly into the target vein [6,7,8,9]. The efficacy of the treatment depends mostly on the type of sclerosing agent and it has improved lately by recent vigorous experimental and clinical trials [10]. The most used sclerotic substances are tetradecyl sulfate sodium (STS) and polidocanol (POL). POL is a non-ionic surfactant with endothelial cell lytic action that solubilizes membrane proteins but does not denature proteins as ionic detergents such as STS. The main advantage of POL is that it is able to lyse platelets at high concentrations but has no destructive effect on plasma-clotting factors preventing the clot formation [11]. The use of foams as drug nanocarriers for POL importantly improves the sclerosing action by improving the delivery in the site of action. Whereas liquid dilutes in the blood upon intravenous injection hence reducing the concentration of drug, foam displaces the blood upon injection, filling the vessel. The bubbles carry the sclerosing agent at the surface, which then comes in full contact with the endothelial cells, maintaining the concentration of drug. The efficacy of foam as a delivery system in sclerotherapy is importantly dependent on the characteristics of the foam—foam stability and bubble size distribution must be adequately controlled. Large bubbles provide high concentrations of air and low available surface area, resulting in lower drug concentration. Conversely, small bubbles decrease the amount of air while increasing the amount of surface area, hence providing higher drug concentrations. However, this size of bubbles needs to be controlled and remain within 10^−6^ m range to prevent solubilization of air into the blood torrent. For this same reason, it is necessary to control foam stability. A poor stability foam can cause decaying the foam to be easily diluted and disseminated through the body so this foam could lead to complications such as pulmonary embolism, deep vein thrombosis, phlebitis, visual disorders, and stroke [12].

Foams are dispersions of gas in liquid media that are produced by application of mechanical energy to create air bubbles. A set of bubbles that are crammed together conforms a foam, which hence comprises large amounts of free surface area. As a consequence, foams are thermodynamically unstable systems that require the use of stabilizing agents [13,14]. These are commonly surface-active molecules such as polymers, proteins, or particles that adsorb onto the air water interface, reducing the surface tension of water and promoting the formation and stabilization of air bubbles [15]. The physicochemical mechanisms governing foam stability are well known and comprise gravitational segregation, coarsening, and drainage of liquid and bubbles’ coalescence [16]. These mechanisms are influenced by different aspects such as the nature of the gas, the size of bubble, polydispersity of the samples, the nature of the liquid phase (viscosity), and by the surface properties of the surface-active molecules in solution [14,16,17].

There are two main lines of action in the literature directed to improve the characteristics (foam stability and bubble size) of current sclerosing formulations: looking at the method of foam formation or the composition of the sclerosing solutions [9]. Concerning the method, there are various techniques available to generate sclerosing foams in the literature [5]. One of the most used techniques is the method of Tessari, which involves mixing the sclerosing solution with room air using a double syringe system connected by a three-way stop cock [18]. Modifications of this method include addition of filters, constrictions [19], or coupling of three syringes [20], while other methods include mechanical agitation [21] or the recent Varithena unit based on pressurized gas [5]. However, the original method of Tessari is still widely applied by medical practitioners and has been extensively used for foam characterization studies [8,22]. In fact, this method has been optimized by evaluating the impact of different parameters on the foam stability. For example, Nastasa et al. established the impact of liquid to air ratio, type of connector, syringe diameter, and number of pumping cycles [23], while more recently by Bai et al. have evaluated multiple factors combined (syringe size, preparation temperature, and pump speed) [12]. The second important parameter influencing the stability of sclerosing foam is the composition the foaming solutions. The first additives tested in the literature deal with increasing the viscosity of the liquid, which is known to improve foam stability by reducing flocculation. For example, Rial et al. addressed the impact of glycerin on foam stability of POL solutions [22], who demonstrated the positive impact on stability. Later, Nastasa et al. evaluated the influence of glycerin and xanthan gum as thickeners, again showing different positive impacts in foam stability, mostly as the viscosity of the liquid increases [8]. These authors also tested the impact of a surfactant (Tween 80), which promoted surface activity of POL. However, all these additives caused an increased size of bubbles in all cases, which can have a negative impact on the performance of the sclerosing foam as explained earlier. This is possibly related to the decreased surface elasticity reported for POL in the presence of additives [13]. More recently, Bai et al. have tested the impact of several surface-active agents (poloxamer 188, Tween 80, macrogol 4000, propanediol, and lecithin) on the stability of sclerosing foam [12]. These again improved the foam stability, but the surface characteristics are not explored therein. Therefore, the presence of POL in the surface of bubbles could be reduced, owing to competitive adsorption. In this regard, the surface properties of the system should be considered along with the stability of foam because they provide information on the composition of the surface of the bubbles. If additives are surface-active, they might compete with POL for the surface of the bubbles, reducing the sclerosing action.

In this work, the additives chosen aimed to look at both stabilizing mechanisms separately. Hence, a surface-active agent (Pluronic-F68, F68) and a thickening agent (hyaluronic acid, HA) are proposed as additives in order to highlight different phenomena. Furthermore, both additives also display bioactive properties, which could provide added functionality to the sclerosing formulations, which constitutes a major advance with respect to previous works. On the one hand, HA is a natural biopolymer present in the extracellular matrix. Owing to its unique biological functions, HA and its derivatives have been explored extensively for biomedical applications such as tissue engineering, drug delivery [24], viscosupplements in orthopaedics, and fillers in the cosmetic industry [25,26]. It is non-surface-active and enhances viscosity in bulk [26]. On the other hand, Pluronic-F68, the commercial name for poloxamer 188, is a non-ionic copolymer triblock with two hydrophilic polyoxyethylene chains (POE) situated in the extremes and one hydrophobic polyoxypropylene chain in the center (POP). It is surface-active and able to self-assemble, forming micelles [27]. It presents numerous applications in pharmaceutical formulations, such as being a stabilizer of cell membranes, emulsifier, solubilizing agent, dispersing agent, and in vivo absorbance enhancer [28] and its biocompatibility with plasma-derived fluids has allowed numerous applications in biomedicine [27,29]. Bai et al. already showed the positive impact of Pluronic-F68 in POL foam [12], however, the specific effect on the surface characteristics of POL remain unexplored, while these are vital in the correct operation of sclerotherapy. Finally, another possible functionality of Pluronic-F68 is its inverse thermosensitivity; it is soluble in aqueous solutions at low temperature, and gels at higher temperature [29]. This special characteristic of Pluronic-F68 could also be interesting in developing thermoresponsive sclerosing foam formulations.

Accordingly, the objective of this research work was to investigate the impact of HA and Pluronic-F68 on the stability of sclerosing foam formed by POL and correlate the effects in the microstructure of foam with the surface properties of the system. Firstly, the method of Tessari for foam production is standardized and automatized. Secondly, the produced foam is characterized by measuring foam stability and bubble size distribution, validating the parameters for its use in sclerotherapy. Finally, a complete surface characterization is shown, providing decisive information on the physicochemical mechanisms underlying the characteristics of sclerosing foam. Results demonstrate that, beyond the stability of foam formulations, the surface composition of bubbles modulates the targeted delivery of polidocanol. The principles evaluated in this work can serve as a guide for evaluating the influence of additives/factors on foam stability, enabling a controlled design of foams as delivery systems in pharmaceutics.

## 2. Materials and Methods

### 2.1. Materials

Thesit (CAS number 9002-92-0, from Sigma-Aldrich, Darmstadt, Germany) is a polyethylene glycol ether of lauryl alcohol, also called polidocanol (POL), and was used as received. POL is a non-ionic surface-active agent consisting of two components: a polar hydrophilic (dodecyl) head and an apolar hydrophobic (polyethylene oxide) chain. This emulsifier is a common cosmetic product, also called Laureth-9, and is used in shampoos and hair conditioners, as well as in body and face creams [11].

Hyaluronic acid (HA) was purchased from Bioiberica S.A.U. (sodium hyaluronate intraarticular high molecular weight, code F0142, batch number 15/0001—1198.486 Da) and was used as received. It is a high molecular weight non-sulfated glycosaminoglicate formed by repetitions of the disaccharide N-acetylglucosamine and N-glucuronic acid.

Pluronic-F68 (CAS 9003-11-6) was purchased from Sigma-Aldrich P1300. Poloxamers are synthetic copolymers that have a hydrophobic block of poly(propylene oxide) (b) surrounded by two hydrophilic blocks of poly(ethylene oxide) (a). Pluronic-F68 is a non-ionic surfactant and its chemical structure has a = 8 and b = 27 [29].

NaCl (CAS number 7647-14-5, from Sigma) was used to prepare 0.9% NaCl, mimicking the physiological solution used by medical practitioners. Ultrapure water, cleaned using a Milli-Q water purification system (0.054 μS), was used for the preparation of all solutions. All glassware was washed with 10% Micro-90 cleaning solution and exhaustively rinsed with tap water, isopropanol, deionized water, and ultrapure water in this sequence.

### 2.2. Methods

#### 2.2.1. Double Syringe Method

**Figure 1:**
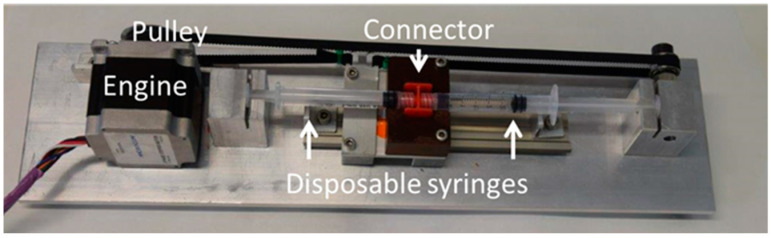
In house double syringe method used for foam designed at the University of Granada and manufactured by Producciones Científicas y Técnicas S.L. (Gójar, Spain).

A double syringe method built in-house was used to generate foam on the basis of the method of Tessari (Figure 1). This prototype was designed at the University of Granada and manufactured by Producciones Científicas y Técnicas S.L. (Gójar, Spain). It consists of two plastic syringes of the same type (3 mL) connected by a sterile luer lock-to-luer lock connector. One syringe contains the sclerosing solution and the air and the other is empty. The two syringes are located facing each other and are moved automatically by means of a pulley, which moves the pistons of the syringes mixing the liquid and the air. The number of cycles can be set, and the rate is maintained constant over time. In this study, the conditions fixed by Nastasa et al. as standard 1:4 liquid/air ratio and 50 pump cycles were applied [23]. After 50 rounds of push and pull, the three-way switch is turned off and the foam is immediately removed from the device and placed on a horizontal desktop (vertical rack). The stability of the foam was quantitatively analyzed by monitoring the height of the foam directly in the syringes as a function of time. The stability of foams was assessed by the foam half lifetime, as the time, in seconds, needed for half of the original volume of sclerotic solution to revert to liquid state. The different mixtures evaluated were diluted in 0.9% NaCl and all the experiments were made at room temperature. The concentrations used in mixtures were: [POL] (0.01–3%), [HA] (0.1–0.5%) and [F68] (10^−5^–10^−1^ mM). Additives were finally fixed to [HA] = 0.3% and [F68] = 0.1 mM and tested over [POL] = (0.01–3%). All the measurements were carried out in triplicate for independent foam samples and final values are plotted as mean values of replicates, with standard deviations. Significant differences between samples (*p* < 0.05) were found according to statistical analysis tools (see Section 2.2.6).

#### 2.2.2. Microscope Images

The bubble distribution in the resulting foam was imaged by optical microscopy using a MOTIC B3 Professional Series microscope. Foams were prepared with the double syringe method (Figure 1) using the same protocol as for the foam stability measurements (1:4 liquid/air ratio, 50 pump cycles, 0.9% NaCl), and the bubble size distribution was measured after 1 min of production. Glass cavity well microscope slides were used as solid support. The well (0.05 mL) was filled with the freshly prepared foam and covered with a glass slide before imaging. Foam images were taken from 5 independent freshly prepared foam samples. The Arc Soft ShowBiz program was used to record the images at different times, and the ImageJ program was used to provide the bubble size distribution. Every image was converted in greyscale, threshold adjusted, and made binary. ImageJ provides the perimeter of the bubbles present in the image its average value. The mean diameter of the bubbles in the foam sample was calculated then as an average from the perimeter by using a known caliber. All the measurements were carried out in triplicate for independent foam samples and the average of the diameter of the bubbles and their deviation showed significant differences between samples (*p* < 0.05) according to statistical analysis tools (see Section 2.2.6).

#### 2.2.3. Pendant Drop Surface Film Balance (DINATEN)

The surface characterization was performed using a pendant drop surface film balance fully designed and assembled the University of Granada (WO2012080536-A1) and described elsewhere [30]. The whole of the equipment is computer-controlled by software DINATEN and the images are analyzed by the software CONTACTO [31]. The detection and calculation of surface area (A) and surface tension (γ) are based on axisymmetric drop shape analysis (ADSA). A solution droplet is formed at the tip of the double capillary and the γ is measured in real time at constant A (20 mm^2^) for 1 h. Then, the dilatational viscoelastic modulus E of the surface adsorbed layer was measured by oscillating of the drop volume. E is a complex quantity that contains a real (E′) and an imaginary part (E″). E′ is the storage modulus that accounts for the surface elasticity (ε) of the adsorbed layer, E″ is the loss modulus that accounts for the surface viscosity (η) of the surface layer: (E = E′ + iE″ = ε + iηε), and ν is the angular frequency of the applied oscillation. The applied volume oscillations are maintained at amplitude values of less than 5%, to avoid excessive perturbation of the interfacial layer and the frequency is varied (0.01, 0.1, and 1 Hz). The pendant drop is kept in a glass cuvette (Hellma) located in a thermostatically controlled cell adjusted to 20 °C with an external temperature control. The surface tension of the clean air–water surface (γ_0_) was measured before every experiment to confirm the absence of surface-active contaminants, yielding values of (72.8 ± 0.2) mN/m at 20 °C. The concentrations of POL used in the surface characterization ranged between 10^−6^ and 0.3% diluted in 0.9% NaCl. These were measured in the absence and presence of 0.3% HA and 0.1 mM F68, separately. All the measurements were carried out in triplicate and the deviation was found to be less than 2%; final values are plotted as mean values of replicates, with standard deviations calculated according to statistical analysis tools (see Section 2.2.6).

#### 2.2.4. Langmuir Film Balance

Langmuir monolayers were measured using a commercial Langmuir film balance equipped with a paper Wilhelmy plate pressure measuring system from KSV (Biolin Scientific, Espoo, Finland) with a sensitivity of 0.1 mN and a total area of 244.5 cm^2^. The whole setup was located in a transparent Plexiglas case to avoid air streams and dust deposition, and the Langmuir trough was connected to a thermostat. The system records the surface pressure-area (π-A) isotherms by measuring the evolution of the surface pressure (π) as the available area (A) is compressed. The π is defined as π = γ_0_ − γ, with γ_0_ being the surface tension of the pure water surface, and γ the surface tension of the monolayer. The absence of surface-active contaminants was verified by compressing the bare water subphase, obtaining values of π < 0.2 mN/m within the whole compression cycle. Then, the POL solution (0.5 g/L) was carefully spread on the subphase by means of a micropipette. After an equilibration time of 15 min, the SP-A isotherm was recorded upon symmetric uniaxial compression at a constant rate of 10 mm/min. Different amounts (10–200 µL) of POL solution were spread on the surface to cover the whole compression isotherm at 20 °C. The POL was spread on pure water subphase and on a subphase containing 3 × 10^−3^ g/L HA. π-A isotherms were carried out in triplicate for both systems and the deviation was found to be less than 2% and final values are plotted as mean values of replicates, with standard deviations calculated according to statistical analysis tools (see Section 2.2.6).

#### 2.2.5. Brewster Angle Microscopy

The morphology of the POL films at AW surface was observed by Brewster angle microscopy using a KSV NIMA MicroBAM (Biolin Scientific, Espoo, Finland) mounted on the Langmuir trough. The MicroBAM is equipped with a 50 mW laser emitting p-polarized light of 659 nm wavelengths and the spatial resolution is 12 µm. The dimensions of each image are 3.6 mm × 4.0 mm. The technique is sensitive to the density and thickness of the film on the air–water surface. No reflected signal is detected on the bare air–water surface at the Brewster angle. The reflect index changes when a monolayer is formed on the surface and thus the light is reflected and detected by the camera [32,33]. MicroBAM images were taken while recording the π-A isotherms of POL and POL + HA. The images shown are representative of at least three independent experiments using three samples prepared separately.

#### 2.2.6. Statistical Analysis

Statgraphics 18 (Statistical Graphics Corp., Rockville, MD, USA) was used for data analysis. Data were expressed as mean ± standard deviation. Firstly, multiple sample comparison analysis (ANOVA) was performed to identify significant differences between samples. Differences between means were considered significant at *p* < 0.05.

## 3. Results

### 3.1. Foam Stability and Bubble Structure

**Figure 2:**
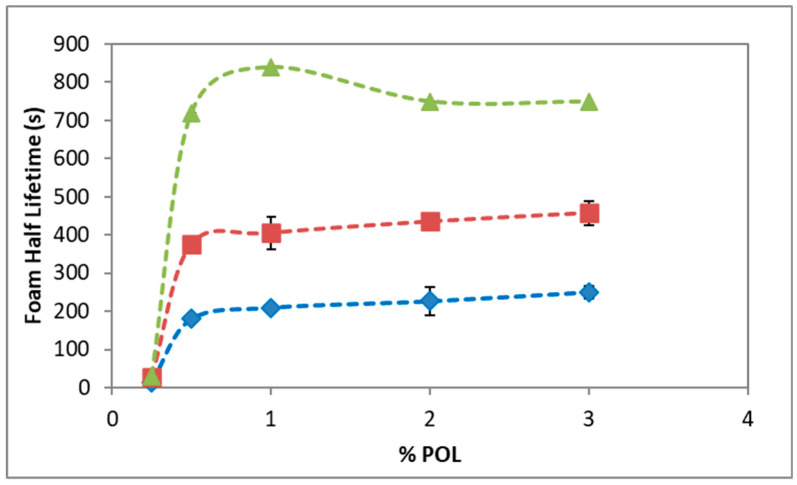
Foam half lifetime of foaming solutions of polidocanol (POL; blue rhomboids), POL + hyaluronic acid (HA) 0.3% (red squares), and POL + Pluronic-F68 (F68) 0.1 mM (green triangles). Foam samples were produced with the double syringe method standardized protocol (1:4 liquid/air, 50 cycles, 0.9% NaCl, and 20 °C). Values plotted are mean values of three independent measurements, and standard deviations according to statistical tools showed significant differences between all samples (*p* < 0.05). Lines are a guide for the eye.

Figure 2 shows the stability of foams of POL, POL + HA, and POL + F68 solutions, as measured by the foam half lifetime, as a function of POL concentrations. The stability of POL foam increased very steeply within the range of POL concentration used. The obtained results for POL agree with those reported in the literature in similar conditions [8,34]. A concentration below 0.1% POL did not produce any foam at all (see Figure S1A) but a slight increase in the POL concentration used, providing a significant improvement in the foam half lifetime. Figure 2 shows that the stability of POL foam attained a pseudo plateau after 0.5%, providing a foam half lifetime of 200–250 s in close agreement with literature [8].

Figure 2 also shows the foam half lifetime of mixtures of POL with 0.3% HA and POL with 0.1 mM F68. The concentrations chosen for HA and F68 were the minimum that provided a noticeable effect in the mixture (see Figure S1B,C). Figure 2 shows that the shape of the foam half lifetime of the mixtures was similar to that of pure POL, but in both cases the foams formed in the presence of additive importantly improved its stability. Namely, the values for foam half lifetime practically doubled that of POL, ranging between 390 and 480 s for the POL + HA mixture and quadruplicated for POL + F68 (720–840 s). This positive impact in stability of POL solutions is similar to that reported by Nastasa et al. using xanthan gum, which has similar rheological properties to HA [8,22]. The impact of F68 in the stability of foam was higher than that of HA. Results from Figure 2 regarding POL + F68, agree with those reported by Bai et al. with the method of Tessari using pure CO_2_, ratio 1:4, 10 rounds of push and pull, and 6% g/mL F68, which corresponded to 370 mM [12]. Interestingly, a similar impact on stability is shown in Figure 2 but using 0.1 mM F68 (Figure 2).

**Figure 3:**
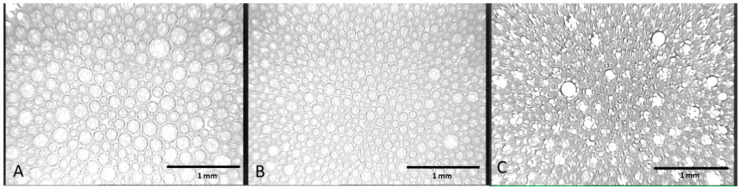
Microscopic scale bidimensional image of foams formed by (**A**) 3% POL, (**B**) 3% POL + 0.3% HA, and (**C**) 3% POL + 0.1 mM F68. Foam samples were produced with the double syringe method standardized protocol (1:4 liquid/air, 50 cycles, 0.9% NaCl, and 20 °C), and the images were taken after 1 min.

Figure 3 shows the bubble size distribution in foams formed by POL, POL + HA, and POL + F68 by means of optical microscopy. The appearance of the bubbles and the foam structure was very peculiar in all cases. The formation of a double foam system can be seen, with the presence of smaller bubbles located inside of larger bubbles (Figure 3A,B). This structure was possibly similar to that of double emulsions. In fact, the spontaneous formation of double emulsions with POL have also been reported recently by Dinache et al. [35]. They mixed in a proportion 1:1 STS 10% solution and vitamin A oily solution (20 mg/mL) by a double syringe diluter-dispenser. Dinache et al. reported the formation of larger drops containing smaller droplets as observed by optical microscopy, and demonstrating the generation of multiple emulsions O/W/O and W/O/W. The double syringe method of foam formation was possibly responsible for the spontaneous formation of a double dispersed phase. Further work on the hydrodynamics of mixing induced by the double syringe method should be accomplished to further explore this phenomenon.

Concerning the impact of additives on the structure and bubble size, we calculated the mean bubble size of POL by ImageJ, providing a mean diameter of D_POL_ = (52 ± 10) µm (Figure 3A). This corresponded to the outer diameter of larger bubbles, which can be clearly outlined. This value correlated with that reported for POL under similar conditions in the literature [8]. Similarly, ImageJ provided a D_POL+HA_ = (42 ± 4) µm for foams made from POL + HA mixtures (Figure 3B), again corresponding to the larger outer bubbles, which can be clearly outlined. This result implies that the presence of HA in the foaming solutions slightly reduces the mean diameter of bubbles while retaining the overall bubble structure of a double foam obtained for POL. The statistical study (ANOVA) showed a *p* < 0.05. Figure 3A,B displays a remarkably similar distribution of double foams composed of larger bubbles containing smaller ones. The slight reduction in mean diameter caused by HA constituted an important advance, contrasting with results obtained in the literature with a similar additive such as xanthan gum, which slightly increased the mean bubble diameter [8]. Even a slight reduction of the size of bubbles can have a positive impact in the performance of the sclerosing foam by increasing the total surface area, and hence the concentration of POL reaching the target. Furthermore, the polydispersity of the bubble distribution, as measured by the standard deviation, is reduced significantly for POL + HA. These findings are clearly connected with the enhanced foam stability of POL + HA foams in Figure 2, as will be discussed in detail later. The molecular origins of this behavior will be analyzed later by evaluating the surface properties of the system. The structure of the foam obtained in the case of POL + F68 (Figure 3C) was somehow different with respect to POL (Figure 3A). It was not possible to measure the diameter for POL + F68 since these images could not be processed by ImageJ to obtain a realistic bubble diameter. The image appeared very dense and difficult to outline (Figure 3C). The inner bubbles appeared less defined and even deformed in the borders, and the sample showed coexisting double foam structures and layered bubbles showing multi thin films surrounding bubbles. More sophisticated tools would be needed to analyze the structure of these bubbles.

### 3.2. Surface Tension

**Figure 4:**
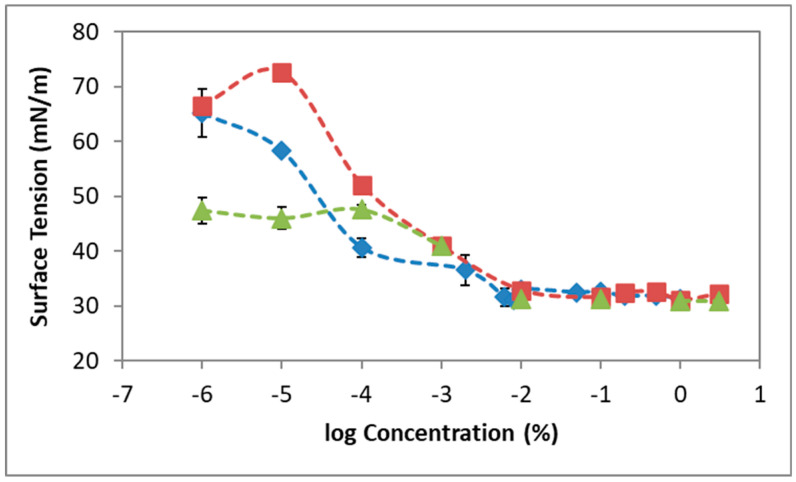
Surface tension values after 1 h of adsorption at constant surface area, 0.9% NaCl and T = 20 °C as a function of bulk concentration of POL. POL (blue rhombus), POL + 0.3% HA (red squares), and POL + 0.1 mM F68 (green triangles). Values plotted are mean values of three independent measurements, and standard deviations are plotted as error bars. Detailed statistical analysis of data is shown in Figure S3. Lines are a guide for the eye.

The creation of foam requires the formation of bubbles; this process is importantly influenced by the surface properties of the system, and hence the surface characteristics of POL, POL + HA, and POL + F68 adsorbed layers were studied in order to correlate with the results obtained on foam stability. Figure 4 shows the final surface tension (γ) attained after 1 h of adsorption of POL, POL + HA, and POL + F68 at constant surface area as a function of the concentration of POL in the mixture. Dynamic curves are shown in the Supplementary Material (Figure S2). Figure 4 shows that the surface tension of POL decreased with concentration in a stepwise γ-c isotherm between 70 and 30 mN/m. The γ-c isotherm showed three regions/adsorption regimes. For [POL] < 10^−4^%, the surface tension decreased to a first plateau; for 10^−4^% < [POL] < 10^−2^%, the surface tension decreased again to a second plateau; and for [POL] > 10^−2^%, the surface tension remained constant. Only few works have explicitly reported the surface characteristics of POL solutions, and there is some variability in the reported data. Most of the works have only reported the surface tension values of the concentrations used by practitioners (0.5–3%) [11,36]. For instance, our values coincided with those reported by Nastasa et al. (around 30 mN/m) for the saturated surface [8], whereas they differed from those reported by Rial et al. (around 55 mN/m) for the saturated surface under similar conditions [22]. Wong et al. recently reported for the first time a γ-c isotherm of POL, obtaining values of surface tension ranging between 40–25 mN/m [34]. López-Cervantes et al. also showed the γ-c isotherm of lauryl ether ethoxylates (C_12_E_9_), obtaining values of surface tension ranging between 50 and 30 mN/m [37]. Accordingly, none of them displayed the lower concentration region with the stepwise behavior. Another interesting parameter to analyze in Figure 4 is the critical micelle concentration (CMC) of a surfactant solution. This is the concentration at which the molecules tend for form aggregates (micelles) in the bulk and can be estimated from the concentration of solution, which provides a saturated surface layer above which the surface tension remains constant. The CMC of POL can be graphically estimated from Figure 4, obtaining 0.0040 ± 0.0005%, which was just slightly lower than that reported in the literature (0.1 mM) [34,37].

The surface tension recorded for the mixed POL + HA system showed a significant effect of HA in the surface properties of POL (Figure 4). The γ-c isotherm of POL + HA showed a monotonous shape with only two regions/adsorption regimes (Figure 4). The surface tension of POL + HA decreased monotonously as a function of POL for [POL] < 10^−1^% and attained a plateau for [POL] > 10^−1^% POL. Interestingly, both curves (POL and POL + HA) overlapped in all the interval of concentrations measured, except in the second regime of the isotherm recorded for POL (10^−3^% < [POL] < 10^−2^%) where the value of surface tension of POL + HA appeared significantly higher than that of pure POL. For higher concentrations, the γ-c isotherms again overlapped in a second plateau, with no significant differences according to statistics. Details on statistical analysis are shown in Figure S3. The CMC value estimated graphically for the mixture POL + HA was (0.0013 ± 0.0005)%, which corresponds to 0.02 mM, only slightly lower than that of POL but significantly different according to statistical analysis. Studies in the literature also reported a slight increase in surface tension of POL caused by xanthan gum and glycerin [8,22]. Non-surface-active polysaccharides (such as xanthan gum and guar gum) can increase the surface tension of surfactants/proteins, owing to the high bulk viscosity and/or the formation of non-surface-active complexes resulting from hydrophobic interactions [38].

The γ-c isotherm obtained for the mixed POL + F68 system appeared different to that of POL and POL + HA, showing the classical behavior of mixtures of non-ionic surfactants. Poloxamers are surface-active agents that compete for the available surface area with POL, as described in the literature with other surface-active compounds [27]. The surface tension behaves like F68 at low concentrations of POL and like POL as the concentrations of POL increases in the mixture. For [POL] < 10^−4^%, the surface tension attained corresponded to the surface tension of F68 at this concentration (0.1 mM), which was lower than that of POL. For [POL] > 10^−4^%, the surface tension attained corresponded to the surface tension of POL, which predominated at the surface. At c = 10^−4^%, the mixture showed a maximum value of surface tension, possibly due to a negative synergism by complexation of POL and F68 at the surface. For higher concentrations ([POL] > 10^−1^%), the γ-c isotherms again overlapped, with no significant differences in the CMC according to statistical analysis (Figure S3).

### 3.3. Surface Dilatational Elasticity and Viscosity

**Figure 5:**
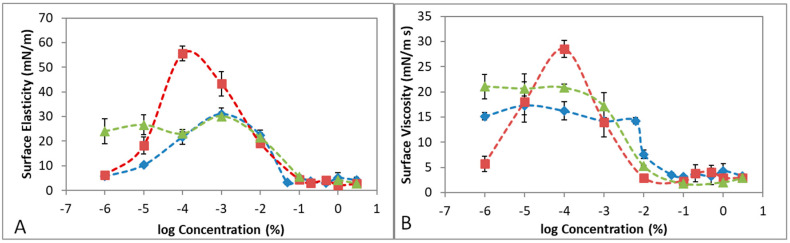
(**A**) Surface dilatational elasticity (1 Hz). (**B**) Surface dilatational viscosity (0.01Hz) attained after 1 h of adsorption at constant surface area, 0,9% NaCl and T = 20 °C as a function of bulk concentration of POL. POL (blue rhombus), POL + 0.3% HA (red squares), and POL + 0.1 mM F68 (green triangles). Values plotted are mean values of three independent measurements, and standard deviations are plotted as error bars. Lines are a guide for the eye.

In order to explain the foam stability, it is useful to evaluate the response of an interface to a perturbation of its adsorbed stable layer [8,13,16]. The dilatational modulus E provides structural information about adsorbed interfacial layers [39], quantifying the ability of the monolayer adsorbed to resist external disturbances and prevent the rupture of the layer [14,16]. E contains information about inter- and intra-molecular interactions of the adsorbed layer, enabling the unravelling of the composition of the surface layer. E is a complex quantity containing the surface elasticity (storage part) and surface viscosity (loss part) of the adsorbed layer. The relative values of the surface elasticity and viscosity depend on the frequency of the oscillation applied [16,39]. Namely, when the oscillation frequency is low, the adsorbed layer has time to adapt to the deformation, allowing for a relaxation process and offering low resistance to deformation (high surface viscosity). Conversely, when the oscillation frequency is high, the adsorbed layer has no time to adapt to deformation and the system resists the compression, behaving as if it was insoluble (high surface elasticity) [16]. Hence, at low frequencies, the surface viscosity of the adsorbed layer is maximized whereas at high frequencies the surface elasticity of the adsorbed layer prevails. Accordingly, in order to study the complete dilatational behavior of POL, POL + HA, and POL + F68 adsorbed surface layers, Figure 5 shows the surface dilatational elasticity values recorded at high frequency (1 Hz) and the surface dilatational viscosity values recorded at low frequency (0.01 Hz). All other frequencies of POL are shown in the Supplementary Material (Figure S4).

Figure 5 shows the surface dilatational elasticity (A) and viscosity (B) of POL, POL + HA, and POL + F68 as a function of POL concentration obtained after 1h of adsorption at constant surface area. In both cases, the curves overlapped at high bulk concentrations [POL] > 10^−2^%, where the presence of POL dominates the behavior of the surface layer. Above the CMC, the surface dilatational response dropped in all the cases due to the high concentration in the system [16,39]. There were no significant differences, according to standard deviations plotted, above the CMC, and the layer was clearly dominated by POL. However, some differences appeared in the dilute regimens, indicative of different complexation phenomena that are analyzed below. The surface dilatational elasticity (Figure 5A) of POL, POL + HA, and POL + F68 showed one single maximum, which was indicative of the existence of a single conformational transition at the surface layer [40]. The height of the maximum was related to molecular interactions within the monolayer and resistance to deformation [41]. The maximum was located at [POL] = 10^−3^% for POL and appeared at lower [POL] = 10^−4^% upon complexation with HA and also significantly higher. Hence, the POL + HA mixture appeared to be more elastic compared to the single POL adsorbed layer. The origins of this improved elasticity can be found in the existence of hydrophobic interactions between POL and HA in the vicinity of the surface, which increased the interactions within the surface film. Similar behavior has been reported for mixtures of protein and non-surface-active polysaccharides [38]. To our knowledge, Nastasa et al. are the only other researchers that have reported dilatational behavior of POL and additives. However, all their additives (glycerin, xanthan gum, and Tween 20) had a negative impact, reducing the surface elasticity of POL. Hence, results shown for POL + HA in Figure 5 comprises a promising new finding, providing evidence of a new molecular mechanism of interaction between POL and HA at the surface, which improved the elasticity of the adsorbed layer. Concerning the mixture of POL + F68, the surface elasticity changed from that reported in the literature for F68 [27] at low [POL], where F68 dominated the surface, to overlapping with pure POL values, when the POL dominated the surface layer (Figure 5A).

Figure 5B also shows the impact of additives in the surface dilatational viscosity of POL adsorbed layers, showing again some significant differences only for dilute systems. To our knowledge, there have been no studies on the surface viscosity of POL reported in the literature. Figure 5B shows that the surface viscosity decreased from a stable value prior to CMC in the case of POL. A similar behavior was found for POL + F68, but the stable value was significantly higher; this could have an impact on the enhanced stability of the foam (Figure 3). Moreover, the presence of HA seemed to improve the surface viscosity by importantly increasing the value before CMC, possibly owing to the complexation between POL + HA. Surface viscosity appeared useful in terms of identifying different interaction mechanisms occurring at the surface layer. Finally, above the CMC, the surface elasticity remained close to zero in all the systems due to the high concentration in the system [16].

### 3.4. Compression Isotherm and microBAM

**Figure 6:**
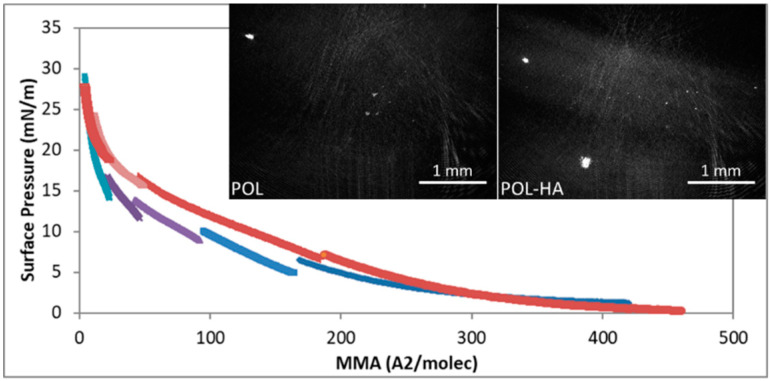
Surface pressure (π)-mean molecular area (MMA) isotherm of POL monolayers in pure water subphase (blue and purple) and in HA 3 × 10^−3^ (g/L) subphase (red and pink). MicroBAM images of POL and POL + HA at π = (12 ± 1) mN/m.

Figure 6 shows the surface pressure–mean molecular area (π-MMA) isotherms obtained for a pure POL monolayer on pure water subphase and on a subphase containing HA (3 × 10^−3^ g/L). The interaction with F68 cannot be assessed with this methodology, owing to the surface activity of F68. POL molecules are soluble, and for this reason, the isotherm was obtained by spreading different amounts of solutions and assembling the curves altogether. Results show that for POL and POL + HA monolayers, the surface pressure increased monotonously, as the area of the monolayer was compressed. At large molecular areas, the POL molecules remained in a 2D gaseous state upon compression of the monolayer until the molecules began to interact. Both isotherms lifted off at similar compression areas, indicating a similar interaction occurring between POL in the absence and presence of HA at low surface densities (π < 5 mN/m). Moreover, both isotherms coincided at the highest compression state (π > 20 mN/m), again indicative of a similar interaction at the highest surface density dominated by POL, in agreement with results from Figure 4 and Figure 5. Conversely, at π values between 5–20 mN/m, the isotherm recorded for POL + HA appeared displaced to higher molecular areas, suggesting an improved interaction at the surface. The increase of the apparent area per molecule could again indicate the existence of hydrophobic interactions between POL and HA in the vicinity of the surface [42], in agreement with the trend obtained in Figure 4 and Figure 5. Moreover, the solubility of the surface layer decreased with HA, as can be seen by the lower number of cycles required to cover the whole compression isotherm. This again could originate a more stable surface film, resulting in more stable foams.

The microBAM allowed us to follow the changes in the morphology of the monolayer as the compression proceeded, as well as the possible effect of HA in the POL monolayer. The contrast in the microBAM images was due to local differences in the monolayer refractive index caused by differences in local molecular density and packing [43]. Dark micro-BAM images were obtained for π < 5 mN/m, indicative of a lack of interaction corresponding to a 2D gas (results not shown). As the π increased in the monolayer, bright spots appeared more numerous for POL + HA, possibly owing to the formation of small aggregates [44]. Apart from the bright spots, large brighter domains appeared, indicative of a higher thickness of the monolayer, indicative of a higher surface coverage attained for POL + HA (Figure 5 POL + HA).

## 4. Discussion

The stability of liquid foams is mainly governed by coarsening, drainage, and coalescence [13,14,16,17]. The first consists in the diffusion of gas from small bubbles to larger ones due to differences in capillary pressure. As a result, the overall size of the bubbles in the foam increases with time. The solubility of the gas in the liquid and the polydispersity of the bubble distribution determine the coarsening of foams. The second destabilizing mechanism, drainage, is mostly related with the viscosity of the liquid separating the bubbles. Finally, coalescence of bubbles is linked to the rupture of the liquid film separating two bubbles to form a bigger one, and hence is strongly dependent on the surface properties of the bubbles, surface tension, and surface elasticity and viscosity. With this in mind, let us analyze the encountered results combining the surface properties and the resulting stability of foam films of POL, POL + F68, and POL + HA.

The stability of the foam produced by POL, using the double syringe method, increased with the amount of POL in the solution until a plateau (Figure 2). The foam half lifetime levels around 200 s were in agreement with works in the literature in similar conditions [8,12,34]. POL is a non-ionic surfactant that stabilized the liquid films by steric forces [34]. As the concentration of POL increased in the foam, the repulsive forces in the liquid lamellae increased, contributing to a disjoining pressure separating the bubbles and favoring the stability of the POL foam. POL is a synthetic fatty acid alcohol (alkyl polyglycol ether of lauryl alcohol). It has a shape of a C, where only one small part of both chains is hydrophobic, and the remainder is hydrophilic [8]. Foam formation is linked to the kinetic adsorption of amphiphilic molecules, which depends on the structure of the molecule, proportion of hydrophobic and hydrophilic groups from the molecule, ionic strength, temperature and pH of the medium, and the concentration [45,46]. Adsorption of POL onto the surface implies the diffusion of POL molecules from the bulk to the surface. Figure S4 shows that increasing POL concentrations provides faster diffusion rates and, in all cases, the surface is rapidly saturated of molecules forming a stable adsorbed layer with different final surface tensions. The final value of surface tension attained is possibly determined by the orientation of the molecule upon adsorption [40,41]. According to Figure 4, the stability of foams appears related to the amount of POL in solution as it decreases the surface tension and hence increases the surface coverage [34,47]. In order to improve the stability of the foam formed by POL for its use as a sclerosing agent for the treatment of varicose veins, we added two biocompatible additives: non-surface-active HA and surface-active F68. HA doubled and F68 quadruplicated the half lifetime of foam for all the ranges of POL concentrations studied.

Mixed solutions of surfactants and less soluble surfactant-active substances are frequently used to improve the stability of foam. The surfactant adsorbs quickly and prevents the foam from collapsing, and the second component adsorbs later and enhances the long-term stability [16]. This is possibly the scenario for amphiphilic molecules agents tested in the literature, such as Tween 80 [8], macrogol 4000, propanediol, lecithin, and poloxamer 188 [12]. In our case, addition of F68 to POL had a strong impact on foam stability. This was due to the double role of F68, having surface activity [27] and increasing bulk viscosity [29]. The surface tension of the mixture approaches that of POL as the concentration of POL increases. This is of vital importance towards the use of this additive in sclerosant treatment since it assures the presence of POL at the surface at sufficient high concentrations of POL in the mixture. F68 should remain in bulk or in a second layer, contributing to the long-term stability. At high POL concentrations, the F68 in bulk improves foam stability by increasing bulk viscosity, which can delay drainage and bubble coalescence. The impact of non-surface-active agents on the foam stability of POL has been less studied in the literature, with glycerin [8,22] and xanthan gum [8] being the additives tested thus far. The lack of surface activity is a positive fact since it guarantees the presence of POL molecules at the surface of bubbles, assuring the performance of the drug. Glycerin and xanthan gum increased the stability POL foam, owing to the increased viscosity of the liquid mixture capable of delaying drainage through bubble interstices [8]. However, in both cases, the bubble size also increased. An increased bubble size was accompanied by a decrease of total surface available, and therefore a reduced amount of POL reaching the target. In fact, Nastasa and coworkers reported a higher surface tension and lower surface elasticity of the mixtures as compared to POL, suggesting that both additives involve lower surface coverage and/or surface cohesion of POL molecules. In this regard, the use of HA provides an important improvement with respect to glycerin and xanthan gum as the increase in foam half lifetime recorded for the mixture POL + HA (Figure 2) was accompanied by a smaller and more mono-disperse bubble size distribution (Figure 3). This implies a higher amount of POL reaching the target as the total surface area increases. Moreover, the improved stability of POL + HA foam can be linked to the bubble size distribution obtained (Figure 3). The bubble size decreased slightly upon addition of HA into the system and, more importantly, the polydispersity of bubble size was significantly reduced, which reduced the impact of coarsening, hence contributing to improvement of the stability of foam obtained. Due to its rheological properties, HA exhibits low viscosity during the pumping cycles (intense mixing process) followed by a larger viscosity after the pumping has stopped [26]. This contributes to a decreased impact of drainage of liquid between bubbles, similar to the action of xanthan gum [8]. Finally, the higher foam half lifetime of POL + HA can be related to a decreased coalescence of bubbles. This depends on film rupture, as determined by the surface properties of the surface layer shown in Figure 3, Figure 4, Figure 5 and Figure 6, which provides evidence of an increased surface coverage and/or surface cohesion of POL molecules in the presence of HA. Higher surface coverage and higher surface connectivity impacts foam stability by preventing film rupture [13,16,47]. Furthermore, the non-surface activity of HA assures the presence of POL at the surface, which is crucial for the optimal delivery of POL.

Application of theoretical models to further understand and predict the stability of foams as a function of the type of additive is another step forward in this research. Moreover, the use of theoretical models to fit the surface tension and the surface elasticity would provide additional information of the surface properties of POL at the molecular level, relevant to the foaming behavior and to the type of interaction occurring between POL and additives. These aspects will be considered in future works. The method of formation also provides routes to improve the stability, as innovative solutions with mechanical agitations will be developed. Finally, the search for new additives that provide added functionality offers a whole new area of research. The use of microgels and nanoparticles, which provide stimuli-responsive systems, are yet to be explored. Finally, these systems also need to be validated in vivo in a future work.

## 5. Conclusions

The use of foam as a delivery system for POL in sclerotherapy is associated with understanding the key factors governing foam stability of sclerosing solutions. Analysis of the surface characteristics of foams provides a new and improved understanding of the physical mechanisms underlying the delivery of POL using foam as a delivery system. HA and F68 constitute new and promising additives in foam sclerotherapy, assuring the presence of POL at the surface while improving foam half lifetime. Our experimental findings enable a direct correlation between foam stability, bubble size distribution, and surface properties of the system. The principles evaluated in this work are proposed as a guide for evaluating the influence of more additives/factors on foam as drug delivery systems. Results shown here demonstrate that, beyond the stability of foam formulations, the surface composition of bubbles modulates the targeted delivery of POL in sclerotherapy.

## Supplementary Materials

The following are available online at https://www.mdpi.com/1999-4923/12/11/1039/s1, Figure S1: Foam stability of POL with additives. Figure S2: Dynamic surface tension of POL. Figure S3: Surface tension isotherms of POL with additives. Figure S4: Surface elasticity and viscosity of POL with additives at different frequencies.
