# Supplementary material for: Effect of Hyaluronic Acid and Pluronic-F68 on the Surface Properties of Foam as a Delivery System for Polidocanol in Sclerotherapy
Source: Pharmaceutics. 2020 Oct 30;12(11):1039. doi: 10.3390/pharmaceutics12111039 (PMC7693533; doi:10.3390/pharmaceutics12111039)
Supplement: Supplementary file 1 [file pharmaceutics-12-01039-s001.docx]

Supplementary Materials: Effect of Hyaluronic Acid and Pluronic-F68 on the Surface Properties of Foam as Delivery System for Polidocanol in Sclerotherapy

Teresa del Castillo-Santaella, Yan Yang, Inmaculada Martínez-González, Maria José Gálvez Ruiz, Miguel Ángel Cabrerizo-Vílchez, Juan Antonio Holgado-Terriza, Fernando Selles Galiana and Julia Maldonado-Valderrama

**
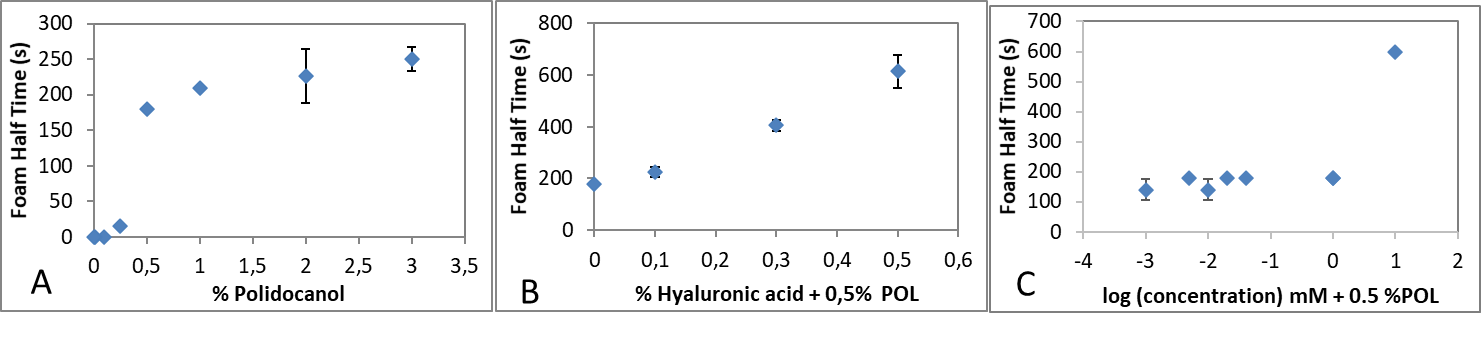
**

**Figure S1.** A. Foam stability of POL. B. Foam stability of HA+ 0.5% POL. C. Foam stability of F68 + 0.5% POL.

Figure S1A shows the foam half lifetime of POL foam obtained with the double syringe method as a function of the concentration of POL at 20 °C and 0.9% NaCl. Stable foams only formed for concentrations >0.5% and a plateau in foam half lifetime rapidly reached. Figure S1B shows the evolution of foam half lifetime as a function of HA in a mixture with a fixed concentration of POL (0.5%). At 0.3% HA the stability of foam was duplicated respect to POL foam but the stabilizing effect increases significantly with the concentration of HA possibly due to enhanced viscosity. Figure S1C shows the evolution of foam half lifetime as a function of F68 in a mixture with 0.5% POL. At 0.1 mM F68 the stability of foam increased three folds the foam half life time respect to the minor concentrations of F68, which showed similar foam half lifetime values to 0.5 % POL (180 s).

**
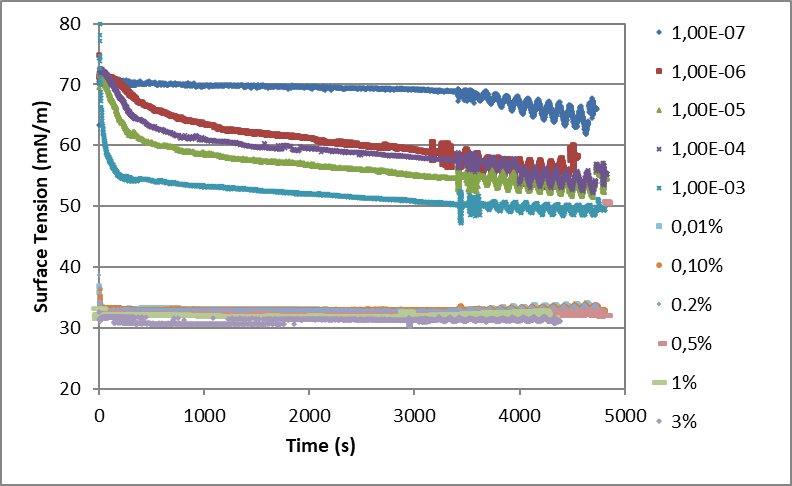
**

**Figure S2.** Representative dynamic surface tension adsorption curves of POL at the air –water interface at a range of concentrations. Dilatational measurements imply the oscillation of the stable layer at the end of the curves. All measurements were made at 20 °C and 0.9% NaCl.

**
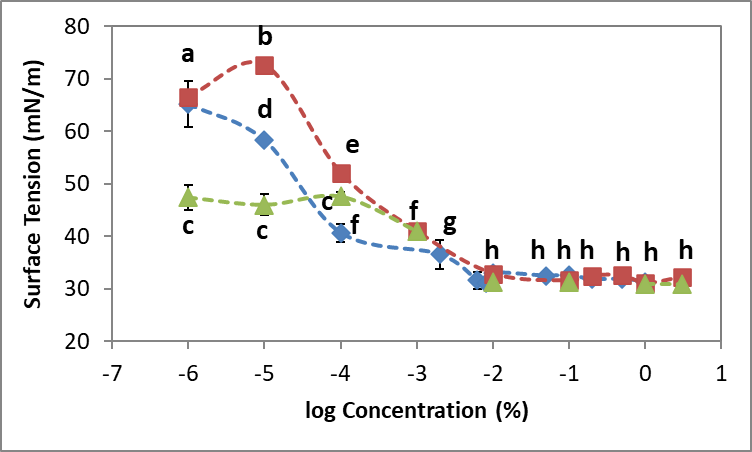
**

**Figure S3.** Surface tension values after 1 h of adsorption at constant surface area, 0.9% NaCl and T = 20 °C as a function of bulk concentration of POL. POL (blue rhombus), POL + 0.3% HA (red squares) and POL + 0.1 mM F68 (green triangles). Values plotted are mean values of three independent measurements and standard deviations according to statistical tools showed significant differences between all samples (*p* < 0.05). Different letters were assigned at values with significant differences.

**
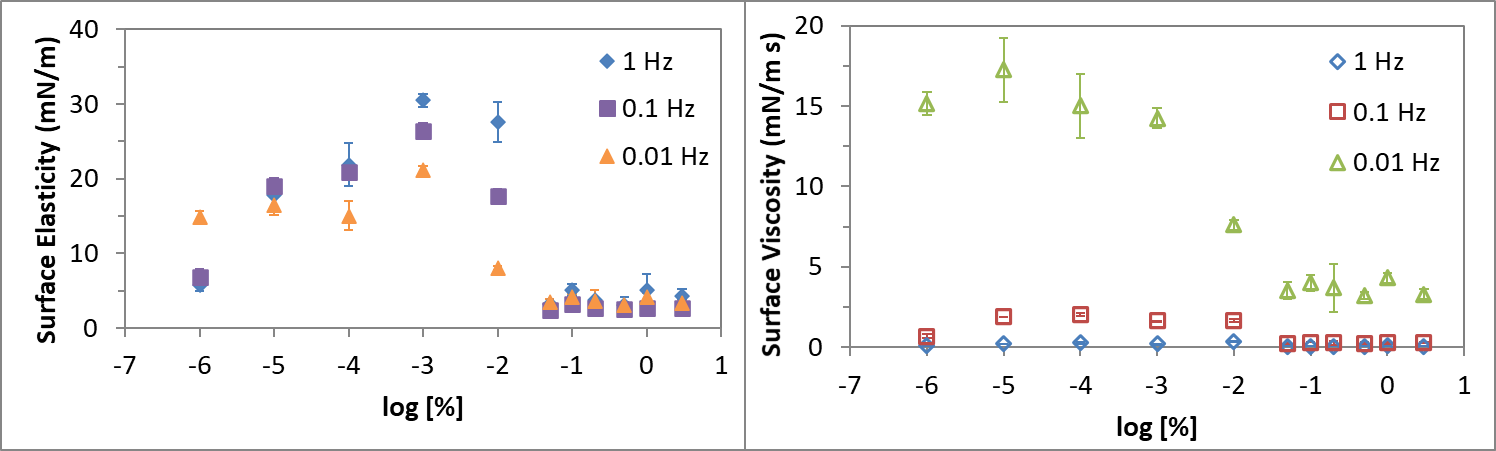
**

**Figure S4.** A. Surface dilatational elasticity. B. Surface dilatational viscosity attained after 1 h of adsorption at constant surface area, 0.9% NaCl and T = 20 °C as a function of bulk concentration of POL at different oscillation frequencies. Values plotted are mean values of three independent measurements and standard deviations calculated according to statistics (section 2.2.6).
